# A single dose of pegfilgrastim compared with daily filgrastim for supporting neutrophil recovery in patients treated for low-to-intermediate risk acute myeloid leukemia: results from a randomized, double-blind, phase 2 trial

**DOI:** 10.1186/1471-2407-8-195

**Published:** 2008-07-10

**Authors:** Jorge Sierra, Jeffrey Szer, Jeannine Kassis, Richard Herrmann, Mario Lazzarino, Xavier Thomas, Stephen J Noga, Nigel Baker, Roger Dansey, Alberto Bosi

**Affiliations:** 1 Division of Clinical Hematology, Hospital de la Santa Creu i Sant Pau, Barcelona, Spain; 2 Department of Clinical Hematology, Royal Melbourne Hospital, Melbourne, Australia; 3 Department of Hematology, Hospital Maisonneuve-Rosemont, Montreal, Canada; 4 Department of Hematology, Royal Perth Hospital, Perth, Australia; 5 Division of Hematology, IRCCS Policlinico San Matteo and University of Pavia, Pavia, Italy; 6 Hematology Service, Edouard Herriot Hospital, Lyon, France; 7 Department of Medical Oncology/Hematology, Alvin and Lois Lapidus Cancer Institute, Baltimore, USA; 8 Biostatistics, Amgen Ltd, Cambridge, UK; 9 Clinical Development, Amgen Inc., Thousand Oaks, CA, USA; 10 Hematology Unit, Department of Critical Care, University of Florence, Florence, Italy

## Abstract

**Background:**

Patients with acute myeloid leukemia (AML) are often neutropenic as a result of their disease. Furthermore, these patients typically experience profound neutropenia following induction and/or consolidation chemotherapy and this may result in serious, potentially life-threatening, infection. This randomized, double-blind, phase 2 clinical trial compared the efficacy and tolerability of pegfilgrastim with filgrastim for assisting neutrophil recovery following induction and consolidation chemotherapy for *de novo *AML in patients with low-to-intermediate risk cytogenetics.

**Methods:**

Patients (n = 84) received one or two courses of standard induction chemotherapy (idarubicin + cytarabine), followed by one course of consolidation therapy (high-dose cytarabine) if complete remission was achieved. They were randomized to receive either single-dose pegfilgrastim 6 mg or daily filgrastim 5 μg/kg, beginning 24 hours after induction and consolidation chemotherapy.

**Results:**

The median time to recovery from severe neutropenia was 22.0 days for both pegfilgrastim (n = 42) and filgrastim (n = 41) groups during Induction 1 (difference 0.0 days; 95% CI: -1.9 to 1.9). During Consolidation, recovery occurred after a median of 17.0 days for pegfilgrastim versus 16.5 days for filgrastim (difference 0.5 days; 95% CI: -1.1 to 2.1). Therapeutic pegfilgrastim serum concentrations were maintained throughout neutropenia. Pegfilgrastim was well tolerated, with an adverse event profile similar to that of filgrastim.

**Conclusion:**

These data suggest no clinically meaningful difference between a single dose of pegfilgrastim and multiple daily doses of filgrastim for shortening the duration of severe neutropenia following chemotherapy in *de novo *AML patients with low-to-intermediate risk cytogenetics.

**Trial registration:**

Clinicaltrials.gov NCT00114764

## Background

Acute myeloid leukemia (AML) is characterized by rapid proliferation of immature clonal myeloid cells, which leads to failure of normal hematopoiesis. Standard treatment for *de novo *AML consists of one or two cycles of intensive induction chemotherapy using cytarabine and anthracyclines with the aim of achieving complete remission. This is followed by one or more cycles of consolidation chemotherapy to maintain remission. Initial remission rates of 60% to 70% are typically achieved in patients aged less than 60 years [1,2].

As a result of their disease, patients with AML are often neutropenic before beginning chemotherapy [3]. Moreover, standard induction and consolidation regimens typically cause profound and protracted neutropenia, with a high attendant risk of infection and death [4,5]. The duration of severe neutropenia is clinically significant, as it is closely correlated with development of infectious complications and associated morbidities [6].

Prophylactic use of myeloid growth factors reduces the severity and duration of neutropenia in patients receiving myelosuppressive chemotherapy [7-9], including patients treated for AML [4,5,10]. Pegylated filgrastim (pegfilgrastim) has the same mechanism of action as the first generation agent filgrastim, but has markedly reduced renal clearance, with neutrophil-mediated clearance being the major route of elimination. As a result, clearance of pegfilgrastim is decreased, and serum concentrations are sustained throughout the duration of neutropenia [11]. A single subcutaneous (SC) dose of pegfilgrastim can provide neutrophil support for chemotherapy regimens with a range of myelosuppressive potential in patients treated for solid tumors [12-14] or lymphoma [15-17], and is as effective as daily SC doses of filgrastim [13,14].

The aim of the current study was to compare the efficacy of pegfilgrastim with filgrastim for assisting neutrophil recovery in patients treated with standard induction and consolidation chemotherapy for AML.

## Methods

### Patients

Patients at least 18 years old with histologically confirmed *de novo *AML, Eastern Cooperative Oncology Group (ECOG) performance status ≤ 2, and life expectancy ≥ 3 months (with treatment) were eligible for study participation. Patients with French American British (FAB) subtype M3 or M7 were excluded. To avoid the inclusion of undetected secondary or postmyelodysplastic syndrome AML, high-risk (unfavorable) cytogenetic disease type AML (-5/del(5q), -7/del(7q), inv(3q), t(3;3), abn 11q23, 20q, 21q, t(6;9), t(9;22), abn 17p, complex karyotypes (≥ 3 abnormalities) [18,19]) were also excluded. Patients with previously treated AML were not eligible for the study.

### Study design

In this randomized, double-blind, multicenter, phase 2 study, patients received a course of standard IA 3+7 induction chemotherapy (idarubicin 12 mg/m^2 ^days 1–3, cytarabine 100 mg/m^2 ^twice daily days 1–7) (Induction 1), with a second course given, if necessary, after neutrophil recovery had occurred (Induction 2). If complete remission was achieved (≤ 5% myeloblasts),[20] patients received one course of high-dose cytarabine consolidation therapy (3 g/m^2 ^if aged <55 years, 2 g/m^2 ^if aged ≥ 55 years or at increased risk for neurotoxicity; administered twice daily over 3 hours on days 1, 3, and 5).

During days 6 through 8 of Induction 1, patients were stratified by age (< or ≥ 55 years) and randomized (using an interactive voice response system [IVRS] and blinded treatment box number assignment) in a 1:1 ratio to receive either pegfilgrastim or filgrastim plus comparator-matched placebo. Separate computer-generated randomization lists were prepared for each age strata. The IVRS allocated the treatments in the order indicated in the list appropriate for the subject's age. Pegfilgrastim (Neulasta^®^; Amgen Inc., Thousand Oaks, CA, USA) was administered as a single SC 6-mg dose approximately 24 hours after completing chemotherapy. Filgrastim (Neupogen^®^; Amgen Inc., Thousand Oaks, CA, USA) 5 μg/kg SC was administered daily beginning 24 hours after chemotherapy and continuing until the post-nadir absolute neutrophil count (ANC) was ≥ 1.0 × 10^9^/L for 3 consecutive days or ≥ 10 × 10^9^/L for 1 day. Patients received the assigned treatment for all courses of chemotherapy. The total treatment duration was up to 3 months with a 1-month follow-up assessment. Patients randomized to active pegfilgrastim also received daily filgrastim-matched placebo injections until recovery of ANC in each cycle. Conversely, patients randomized to active daily filgrastim received a single pegfilgrastim-matched placebo injection in each cycle. Matched placebo vials were indistinguishable from those containing active agent. Both pegfilgrastim and filgrastim were formulated as clear, colorless, aqueous solutions. Placebo formulations contained the same excipients with no active agent.

The primary objective of the study was to compare time to recovery from severe neutropenia for the pegfilgrastim and filgrastim treatment groups. Other objectives were to compare the rate of complete remission following induction chemotherapy, ANC, adverse events, and the incidence and duration of hospitalization, fever, and intravenous anti-infective use.

In order to confirm that pegfilgrastim concentrations were maintained during prolonged neutropenia, a pharmacokinetic substudy was planned for the timepoint when 60 patients had completed Induction 1. The substudy was conducted by Amgen personnel (hematologist/oncologist, biostatistician, safety specialist, and medical affairs director) not associated with the study.

The study was conducted in accordance with the Declaration of Helsinki and with International Conference on Harmonization principles of good clinical practice. The appropriate independent ethics committees or institutional review boards reviewed and approved the protocol and informed consent forms. Written informed consent was obtained from all patients before study-specific procedures were performed.

### Pharmacokinetic analysis

Serum samples for determining pegfilgrastim concentrations were collected concurrently with complete blood count samples in Induction 1 (daily until ANC recovery occurred) and were analyzed using a validated enzyme-linked immunosorbent assay (ELISA). Pharmacokinetic parameters were estimated using noncompartmental analysis of serum concentration-time data.

### Statistical analysis

Statistical methods were descriptive, with two-sided 95% confidence intervals (CI) for treatment differences calculated when appropriate. Time to recovery from severe neutropenia (ANC <0.5 × 10^9^/L) was calculated from the first day of chemotherapy until the first of two consecutive post-nadir ANC values ≥ 0.5 × 10^9^/L. Patients who did not develop severe neutropenia were considered recovered at day 1, and time to recovery was censored (at last ANC value) for patients who withdrew without recovery, started the next cycle before recovery, or did not recover. Summaries of time to neutrophil recovery were derived using Kaplan-Meier methods. Duration of febrile neutropenia (FN) (ANC <0.5 × 10^9^/L and oral temperature ≥ 38.0°C) was calculated only in Induction 1 and defined as the onset time until the first of 2 consecutive days with resolution of both neutropenia and fever, or until the last day of the cycle if both parameters had not recovered. Duration of fever was counted from the first day with an oral temperature ≥ 38.0°C until the first of 2 consecutive days with temperature <38.0°C, or the last day of the cycle if not resolved.

The planned sample size of 120 patients (based on the width of the 95% CI around the difference between the median times to ANC recovery in the two groups) was sufficient to estimate treatment differences with acceptable accuracy. With 60 subjects per treatment group, the half-width of the 95% CI for the difference between treatment groups was estimated to be 2 to 3 days. On the basis of a previous study, it was calculated that the sample size would provide up to 70% power to provide a preliminary conclusion of noninferiority. The full analysis set included all randomized patients who received at least one dose of pegfilgrastim or filgrastim.

## Results

### Patients and study conduct

Between March 2003 and April 2004, 84 patients from 27 investigational sites in Australia, Europe, and North America participated in the trial.

A planned interim analysis (after 60 patients had completed Induction 1) revealed an apparent difference in time to recovery from severe neutropenia between the two treatment groups. In the interests of patient safety, it was decided to suspend the study (ongoing patients discontinued study treatment and no new patients enrolled). However, subsequent review of the data by the study sponsor revealed a statistical programming error in the analysis of neutrophil recovery: the time to ANC recovery had been evaluated using a threshold ≥ 2.0 × 10^9^/L instead of ≥ 0.5 × 10^9^/L (the protocol-defined end point). As patients in the filgrastim arm received treatment until ANC was ≥ 1.0 × 10^9^/L for 3 consecutive days, it was logical that they surpassed 2.0 × 10^9^/L earlier than patients receiving pegfilgrastim. When this programming error was corrected, in the interim analysis the difference in time to ANC recovery between treatment groups was 0.5 days (95% CI: -1.8 to 2.8). This precision was within the boundaries set when the study was planned (2–3 days). After careful statistical consideration, it was decided that the study would not be restarted, as the potential bias introduced by halting the study prematurely was felt to be outweighed by the bias that would have been associated with restarting a partially unblinded study (note, no patients were unblinded). Moreover, the reduced sample size was large enough to estimate with adequate precision any differences between treatment groups with respect to the primary endpoint.

Of 84 randomized patients administered Induction 1 chemotherapy, 83 (42 pegfilgrastim, 41 filgrastim) received study drug and were evaluable for efficacy and safety. Baseline demographic and patient characteristics were similar between treatment groups (Table 1). Forty-six patients (22 pegfilgrastim, 24 filgrastim) received consolidation therapy after achieving complete remission. Because of the small number of patients who received Induction 2 chemotherapy (1 pegfilgrastim, 3 filgrastim), most efficacy data are presented for Induction 1 and Consolidation only.

**Table 1:** Demographics and disease characteristics

	Pegfilgrastim 6 mg	Filgrastim 5 μg/kg/day
No. of Patients	42	41
Age		
Median (range), years	51 (18–74)	54 (19–79)
< 55 years, n (%)	22 (52%)	21 (51%)
≥ 55 years, n (%)	20 (48%)	20 (49%)
Male, n (%)	22 (52%)	17 (41%)
Baseline ANC, × 10^9^/L		
Mean (SD)	2.2 (5.4)	2.3 (4.0)
Median (range)	0.4 (0.0–33.8)	1.0 (0.0–17.4)
Most common FAB Type, n (%)		
M1	9 (21%)	9 (22%)
M2	8 (19%)	11 (27%)
M4	12 (29%)	4 (10%)
M4eo	3 (7%)	3 (7%)
M5	4 (10%)	6 (15%)
Cytogenetics, n (%)		
Intermediate	34 (81%)	38 (93%)
Favorable	7 (17%)	3 (7%)
Unfavorable^a^	1 (2%)	0 (0%)
Bone marrow cellularity, n (%)		
Hypoplastic	4 (10%)	5 (12%)
Normoplastic	4 (10%)	8 (20%)
Hyperplastic	33 (79%)	28 (68%)
Unknown	1 (2%)	0 (0%)

ANC, absolute neutrophil count; SD, standard deviation; FAB, French American British.

^a^Cytogenetic classification re-evaluated after randomization; patient ineligible, but included in analyses consistent with intent-to-treat principles.

Twenty-one patients (50%) in each treatment group completed the study. No significant differences between treatment groups were observed with respect to reasons for early withdrawal. Of 42 patients who were withdrawn prematurely, 15 were discontinued due to the early closure of the study. All but one of these patients (who never received randomized study medication) were included in all analyses, using censored values where appropriate. Only 6 patients (3 per group) had not recovered from severe neutropenia in Induction 1 at the time of study closure; these patients were permitted to receive open-label filgrastim treatment (2 patients in total received additional filgrastim therapy). The impact on the study of these early withdrawals at their relevant timepoints was evaluated in a sensitivity analysis and no effect was seen. In addition to those withdrawn because of study closure, an additional 27 patients were withdrawn from the study prematurely because of: failure to achieve complete remission (2 pegfilgrastim, 3 filgrastim), clinically significant delay (>14 days) in administration of consolidation chemotherapy following remission assessment (4, 1), adverse events (2, 2), death (1, 2), or other reasons (4, 6).

At least 90% of patients received full dose chemotherapy (>75% of protocol-specified dose) in Induction 1 and Consolidation. The median number of filgrastim doses administered was 16 during Induction 1 and 13 during Consolidation.

### Time to recovery from severe neutropenia

**Figure 1:**
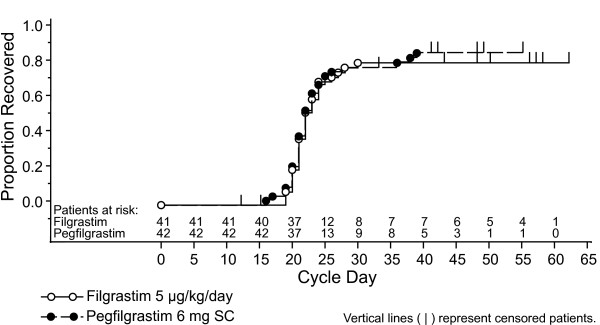
Kaplan-Meier estimates of time to recovery from severe neutropenia in Induction 1.

All patients had severe neutropenia in Induction 1, and ANC recovered in most patients (Table 2). For both treatment groups, the estimated median time to ANC recovery was 22.0 days (difference between groups 0.0; 95% CI: -1.9 to 1.9 days) (Figure 1). Results were also similar when analyzed by age (<55 or ≥ 55 years) and cytogenetic type (intermediate or favorable), with median time to ANC recovery ranging from 22.0 to 24.0 days across subgroups and treatments (data not shown).

**Table 2:** ANC recovery in Induction 1 and Consolidation

	Pegfilgrastim 6 mg (n = 42)	Filgrastim 5 μg/kg/day (n = 41)
Induction 1		
Number of patients starting cycle	42	41
Number of patients (%) with SN	42 (100%)	41 (100%)
Number of patients (%) with ANC recovery^a^	35 (83%)	32 (78%)
Median time to ANC recovery^b^	22 days	22 days
Range	16, 55	19, 62
Difference between medians (95% CI)	0.0 (-1.9 to 1.9)	
Consolidation		
Number of patients starting cycle	22	24
Number of patients (%) with SN	20 (91%)	21 (88%)
Number of patients (%) with ANC recovery^a^	18 (82%)	23 (96%)
Median time to ANC recovery^b^	17 days	16.5 days
Range	1, 57	1, 51
Difference between medians (95% CI)	0.5 (-1.1 to 2.1)	

ANC, absolute neutrophil count; SN, severe neutropenia (ANC <0.5 × 10^9^/L); CI, confidence interval.

^a^Includes patients whose ANC remained at or above 0.5 × 10^9^/L

^b^Number of days from start of chemotherapy until the first of 2 consecutive days with ANC ≥ 0.5 × 10^9^/L; time to recovery was set to 1 day for patients whose ANC remained at or above 0.5 × 10^9^/L

During Consolidation, most patients had severe neutropenia, and again, ANC recovered in most cases (Table 2). Median time to ANC recovery was 17.0 days for pegfilgrastim versus 16.5 days for filgrastim (difference 0.5; 95% CI: -1.1 to 2.1 days). All 5 patients (4 pegfilgrastim, 1 filgrastim) who were classified as ANC recovery failures after Consolidation had a late ANC nadir after receiving additional off-study chemotherapy and were not followed up for sufficient time to document ANC recovery.

### ANC profile and pegfilgrastim pharmacokinetics

**Figure 2:**
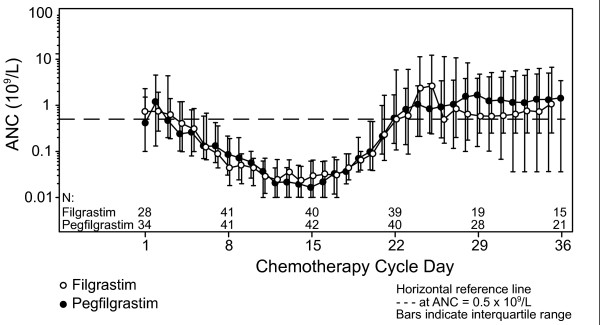
Median absolute neutrophil count for pegfilgrastim and filgrastim recipients in Induction 1.

As shown in Figure 2, median ANC profiles for each treatment group were almost superimposable up to day 21 of Induction 1. A second peak was observed after day 21 in the filgrastim group, who received treatment until ANC recovery.

**Figure 3:**
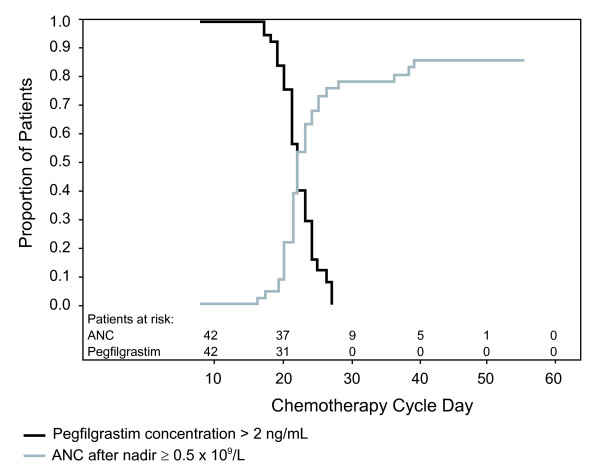
Relationship between days to pegfilgrastim concentration falling below 2 ng/mL and days to absolute neutrophil count > 0.5 × 10^9^/L

After a single dose in Induction 1, median pegfilgrastim serum concentrations reached a maximum (181 ng/mL) 72 hours postdose and remained above the clinically relevant threshold (2 ng/mL, derived from modeling) [21] throughout the prolonged duration of neutropenia (approximately 21 days). Pegfilgrastim concentrations declined on ANC recovery, consistent with a neutrophil-mediated clearance mechanism. A positive correlation (Spearman rank correlation = 0.485, *P *= 0.004) was observed between time to ANC recovery and time to pegfilgrastim concentration falling below 2 ng/mL (Figure 3).

### Febrile neutropenia and fever

Thirty four patients (81%) in the pegfilgrastim group versus 36 patients (88%) in the filgrastim group developed FN, according to the protocol-specified definition, during Induction 1. The median duration (interquartile range) of FN during this phase was 15 (11, 20) days for the pegfilgrastim group, versus 14 (11.5, 18.5) days for the filgrastim group. The incidence and median (interquartile range) number of days with fever were similar in both treatment groups during Induction 1 (90%, 5 (3, 8) days for pegfilgrastim *vs *93%, 6 (3, 12) days for filgrastim). During Consolidation, fever was reported in more patients in the pegfilgrastim group (17/22 (77%)) versus the filgrastim group (14/24 (58%)), but the median duration (interquartile range) was 2 (2, 3; 2, 2) days in each group.

### Complete remission

**Figure 4:**
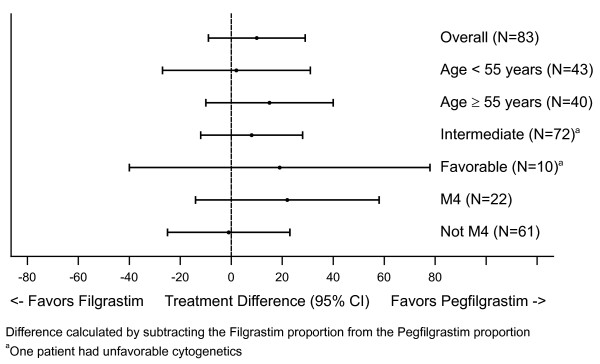
Proportion of patients in complete remission overall and by subgroup.

At the end of Induction 1, 33 pegfilgrastim (79%) versus 26 filgrastim recipients (63%) achieved complete remission. Two additional filgrastim recipients achieved complete remission after Induction 2, resulting in overall complete remission rates of 79% (33/42) in the pegfilgrastim group versus 68% (28/41) in the filgrastim group. No difference in complete remission was observed between treatment groups, overall (95% CI for difference in proportions: -9% to 29%), or when analyzed by cytogenetic type or age group (Figure 4). When rates for patients with FAB subtype M4 (the subgroup with the largest difference between treatment groups) were compared with those having other FAB subtypes, the complete remission rate was higher within the M4 subtype for pegfilgrastim patients, however due to the width of the CIs, no clear differences emerged ("M4:" pegfilgrastim 14/15 (93%) *vs *filgrastim 5/7 (71%); "Not M4:" pegfilgrastim 18/27 (67%) *vs *filgrastim 23/34 (68%)).

### Anti-infective use and hospitalization

Nonprophylactic anti-infectives were administered to all but two patients (both from the filgrastim group) during Induction 1; the median duration (interquartile range) of use was lower among pegfilgrastim (18.5 (13, 24) days) versus filgrastim recipients (21 (14, 27) days). During Consolidation, nonprophylactic anti-infective use was higher in the pegfilgrastim (82%) versus the filgrastim (67%) group. However, the median (interquartile range) duration of use was similar in the two groups (pegfilgrastim 21 (20, 33) *vs *filgrastim 21.5 (18, 35.5) days). The incidence and duration of hospitalization was similar in the two treatment groups, with nearly all patients being hospitalized, as per routine clinical practice.

### Adverse events

All 83 patients had one or more adverse event. The pattern of events was consistent with that expected in a population receiving intensive chemotherapy for AML and was similar for the two treatment groups. Treatment-related adverse events occurred in 11 (26%) pegfilgrastim versus 9 (22%) filgrastim recipients. One of these events (vascular purpura; pegfilgrastim group) was classified as serious. The most frequently reported treatment-related adverse event was bone pain (pegfilgrastim 3 (7%) *vs *filgrastim 4 (10%)). Two patients (5%) withdrew from each treatment group as a result of adverse events. Three patients died during the study (1 pegfilgrastim, 2 filgrastim), all of respiratory complications associated with chemotherapy toxicity and baseline co-morbid conditions. None of the deaths were considered related to study drug.

## Discussion

This randomized, double-blind phase 2 study was the first to evaluate use of pegfilgrastim for neutrophil support in patients receiving chemotherapy for AML. Few studies on the use of pegfilgrastim in this setting have been published and none address the efficacy of pegfilgrastim in relation to filgrastim.

Treatment was anticipated to induce profound and protracted neutropenia with a high risk of FN. The efficacy and safety of filgrastim in this setting was previously demonstrated in a large (n = 521), randomized, double-blind trial, which showed significant reductions compared with placebo in the duration of severe neutropenia (*P *< 0.001) and fever, anti-infective use, and hospitalization [4]. In the present study, we found no evidence to suggest a clinically meaningful difference between the efficacies of a single dose of pegfilgrastim 6 mg or daily filgrastim 5 μg/kg for reducing time to recovery from severe neutropenia – the primary endpoint. More than 80% of both treatment groups developed FN during Induction 1. FN was somewhat prolonged in this phase (median 15 days for pegfilgrastim *vs *14 days for filgrastim), but this probably reflects the predefined stipulation for resolution of both fever and neutropenia before the event was considered resolved. Indeed, the duration of fever was much shorter than this (median 5 days pegfilgrastim *vs *6 days filgrastim) and correlates well with data previously reported in this setting (7 days filgrastim *vs *8.5 days placebo) [4].

Serum pegfilgrastim concentrations were sustained throughout the prolonged period of severe neutropenia and declined rapidly on ANC recovery. A positive correlation was observed between time to ANC recovery and time to pegfilgrastim concentration falling below the therapeutic threshold, further supporting the neutrophil-mediated clearance mechanism for pegfilgrastim. Pegfilgrastim was well tolerated and the adverse event profile was comparable to previously published data on filgrastim use in AML [4].

Initial complete remission rates in our study were similar between treatment groups and comparable to results from previous studies in this setting [4,22-25]. Correlation between remission induction and improved survival was clearly demonstrated by Heil et al [4]: median survival times for patients achieving versus not achieving complete remission were 18.2 versus 4.4 months. Concerns that hematopoietic growth factors might stimulate growth of the myeloid leukemic clone in patients have not been confirmed in clinical studies: to date, leukemic clone stimulation has been demonstrated only *in vitro *[26-28]. Long-term follow up (median 7 years) of patients in the Heil et al. study showed that filgrastim treatment did not have any adverse effects on complete remission or long-term survival rates [29]. Since filgrastim and pegfilgrastim have the same active moiety, we would expect long-term outcomes for pegfilgrastim to be consistent with those for filgrastim.

Patients with unfavorable cytogenetics or secondary, relapsed, or previously treated AML were excluded from our study. It is known that neutrophil recovery may be delayed in such patients, and their exclusion was intended to minimize heterogeneity with respect to duration of severe neutropenia and thereby allow precise evaluation of the impact of the growth factors on neutrophil recovery time in the two treatment groups. The power of this study was weakened by early termination of recruitment and by patient withdrawals. The impact of study closure on the primary endpoint was balanced between treatment groups, as the same number of patients were withdrawn from both groups prior to recovery from severe neutropenia in Induction 1. This was a phase 2 trial and no specific definition of non-inferiority was planned, instead the study was designed to have sufficient subjects to estimate the difference between the groups in time to neutrophil recovery within ± 2–3 days, which was predefined as the period pertaining to a clinically relevant difference. Despite early termination of the study, which resulted in reduced patient numbers, the 95% CI observed (± 1.9 days) was narrower than that constituting a clinically relevant difference in neutrophil recovery between pegfilgrastim- and filgrastim-treated patients. Within this context, our findings provide no evidence of a clinically relevant difference between the treatment strategies. Data regarding secondary endpoints must, however, be interpreted within the limits of the revised sample size. Overall, given the paucity of data in this setting, the current study provides valuable information and an evidence base for the use pegfilgrastim in AML.

## Conclusion

In conclusion, data from this phase 2 study suggest no difference in the efficacy of a single dose of pegfilgrastim compared with daily doses of filgrastim for reducing the duration of severe neutropenia in patients receiving induction and consolidation chemotherapy for low-to-intermediate risk AML.

## Competing interests

RH is a member of an Amgen advisory board. SJN is a consultant to Amgen and is a member of Amgen speaker bureau. NB and RD are employees of Amgen.

## Authors' contributions

JSi was the lead investigator of the study and participated in data acquisition. JSz, JK, RH, ML, XT, SJN and AB were involved in data acquisition. NB carried out data analyses. RD was involved in design of the study and interpretation of data. All authors reviewed the manuscript for intellectual content and approved the final version.

## Pre-publication history

The pre-publication history for this paper can be accessed here:


